# Diaphragmatic Breathing Reduces Exercise-Induced Oxidative Stress

**DOI:** 10.1093/ecam/nep169

**Published:** 2011-02-10

**Authors:** Daniele Martarelli, Mario Cocchioni, Stefania Scuri, Pierluigi Pompei

**Affiliations:** Department of Experimental Medicine and Public Health, University of Camerino, Via Madonna delle Carceri, 62032 Camerino, Macerata, Italy

## Abstract

Diaphragmatic breathing is relaxing and therapeutic, reduces stress, and is a fundamental procedure of Pranayama Yoga, Zen, transcendental meditation and other meditation practices. Analysis of oxidative stress levels in people who meditate indicated that meditation correlates with lower oxidative stress levels, lower cortisol levels and higher melatonin levels. It is known that cortisol inhibits enzymes responsible for the antioxidant activity of cells and that melatonin is a strong antioxidant; therefore, in this study, we investigated the effects of diaphragmatic breathing on exercise-induced oxidative stress and the putative role of cortisol and melatonin hormones in this stress pathway. We monitored 16 athletes during an exhaustive training session. After the exercise, athletes were divided in two equivalent groups of eight subjects. Subjects of the studied group spent 1 h relaxing performing diaphragmatic breathing and concentrating on their breath in a quiet place. The other eight subjects, representing the control group, spent the same time sitting in an equivalent quite place. Results demonstrate that relaxation induced by diaphragmatic breathing increases the antioxidant defense status in athletes after exhaustive exercise. These effects correlate with the concomitant decrease in cortisol and the increase in melatonin. The consequence is a lower level of oxidative stress, which suggests that an appropriate diaphragmatic breathing could protect athletes from long-term adverse effects of free radicals.

## 1. Introduction

Stress is defined as a physiological reaction to undesired emotional or physical situations. Initially, stress induces an acute response (fight or flight) that is mediated by catecholamines. When stress becomes chronic and lasts for a long time, the stressed organism reacts with physiological alterations to adapt to the unfavorable conditions. This ACTH-mediated reaction affects the immune and neuroendocrine systems, and it is responsible for several diseases [1]. Numerous data support the hypothesis that the pathophysiology of chronic stress can be due, at least partially, to an increase in oxidative stress [2–4], which may also contributes to heart disease [5, 6], rheumatoid arthritis [7, 8], hypertension [9, 10], Alzheimer's disease [11, 12], Parkinson's disease [13], atherosclerosis [14] and, finally, aging [15].

Some authors have attributed stress-induced oxidative stress to an increase in glucocorticoids. In fact, there is evidence to suggest that glucocorticoids induce oxidative stress mainly by altering the expression and activity of antioxidant enzymes, thus impairing the antioxidant defense of the body [16–19]. High levels of glucocorticoids are known to decrease blood reduced glutathione (GSH) and erythrocyte superoxide dismutase (SOD) activity in rats [20]. Other enzymes are also involved, and NADPH oxidase, xanthine oxidase and uncoupled endothelial nitric oxide synthase are important sources of reactive oxygen species (ROS) in glucocorticoid-induced oxidative stress (see [9] for a review on this argument).

A number of studies support the fact that meditation, through the modulation of the neuroendocrine response, combats stress and its related diseases. In fact, beyond its psychological and social effects, clinical studies have documented that meditation improves the immune system [21] and decreases cardiovascular risk factors such as hyperlipidemia, hypertension and atherosclerosis [22–30]. A reduction in both glucocorticoids and oxidative stress has been documented in people who practice meditation regularly. Hormonal reactions to stressors, in particular plasma cortisol levels, are lower in people who meditate than in people who do not [31–36], suggesting that it is possible to modulate the neuroendocrine system through neurological pathways. Analysis of oxidative stress levels in people who meditate indicated that transcendental meditation, Zen meditation and Yoga correlate with lower oxidative stress levels [37–43].

Melatonin could also be involved in the reduction of oxidative stress because increased levels of this hormone have been reported after meditation [44–46]. This neurohormone is considered a strong antioxidant and is used as a treatment for aging. Melatonin in fact, increases several intracellular enzymatic antioxidant enzymes, such as SOD and glutathione peroxidase (GSH-Px) [47, 48], and induces the activity of *γ*-glutamylcysteine synthetase, thereby stimulating the production of the intracellular antioxidant GSH [49]. A number of studies have shown that melatonin is significantly better than the classic antioxidants in resisting free-radical-based molecular destruction. In these *in vivo* studies, melatonin was more effective than vitamin E, *β*-carotene [50–52] and vitamin C [53–55]. In addition to mental stress, physical stress also increases the production of ROS. In exhaustive and prolonged exercise, ROS production is elevated, and changes in exercise intensity (aerobic-anaerobic) have been associated with a higher degree of oxidative stress [56–59]. Although it has been established that a continuous and moderate physical activity reduces stress, intense and prolonged exercise is deleterious and needs a proper recovery procedure. The link between physical and psychological stress is apparent because they have equivalent hormonal responses. Actually, both types of stress are characterized by activation of the neuroendocrine axis, which leads to the production of ACTH and cortisol. The beneficial or detrimental role of cortisol in athletes has been debated, as some believe that its catabolic actions are detrimental to muscle recovery, whereas others believe that its anti-inflammatory actions are beneficial to muscle recovery. Plasma cortisol levels increase in response to intense and prolonged exercise [60, 61]. Ponjee et al. [62] demonstrated that cortisol increased significantly in male athletes after they ran a marathon. In another study, plasma ACTH and cortisol were found elevated in highly trained runners and in sedentary subjects after intense treadmill exercise [63].

Additionally, melatonin levels are affected by physical activity. There are some conflicting reports regarding the effects of exercise on melatonin levels, with some studies reporting an increase, some a decrease and some reporting no change in melatonin concentrations after exercise [64–70]. However, these contradictory results could be due to light conditions and the timing or intensity of exercise. Moreover, sex, age and training of the monitored athletes may contribute to the different results reported in these studies. It has been speculated that intense sport increases melatonin secretion due to the necessity of combating the free radical production that occurs during exercise, and melatonin could be responsible for amenorrhea in female athletes as an effect of overtraining [71].

Most, if not all, meditation procedures involve diaphragmatic breathing (DB), which is the act of breathing deeply into the lungs by flexing the diaphragm rather than the rib cage. DB is relaxing and therapeutic, reduces stress and is a fundamental procedure of Pranayama Yoga, Zen, transcendental meditation and other meditation practices.

Although exercise-induced ROS production can be produced via different pathways [56], we speculated that by combating the exercise-induced increase in cortisol levels and by stimulating melatonin levels, DB could improve antioxidant defenses and, therefore, decrease oxidative stress. We have recently demonstrated that in master athletes, oxidative stress induced by intense exercise reaches dangerous levels [72]. Therefore, in this study, we investigated the effects of DB on exercise-induced oxidative stress and the putative role of cortisol and melatonin hormones in this stress pathway.

## 2. Methods

### 2.1. Subjects and Exercise

Athletes were monitored during a training session for a 24-h long contest. This type of race lasts for 24 h, generally starting at 10:00 am and ending at 10:00 am the following day. Bikers ride as many kilometers as possible on a specific circuit trail in the 24-h period. Athletes are allowed to stop, to sleep, to rest and to eat as much food as they want to eat.

The session analyzed in this study was a reproduction of the first 8 h of the race, which is generally the most intense. Athletes started to ride at 10:00 am and stopped at 6:00 pm. They consumed the same food and rested the same time.

Since the parameters measured can differ for each individual, we performed preliminary analyses to select subjects with comparable cortisol, melatonin, antioxidant and oxidative stress values.

We selected 16 amateur male cyclists, aged 44.4 ± 2 years (±SD). Their mean height and weight were 175.4 ± 7.5 cm and 68.8 ± 5.7 kg, respectively, (Table 1). Subjects were informed of the purpose of the study, and all of them gave their informed consent prior to their inclusion. This study has been performed in accordance with the ethical standards laid down in the 1964 declaration of Helsinki.

None of the subjects had taken medication or supplements within the past 30 days that might alter the study outcome, and none of them had a history of medical or surgical events that could affect the study outcome, including cardiovascular disease or metabolic, renal, hepatic or musculoskeletal disorders.

### 2.2. Experimental Procedure

After exercising, athletes took a shower and drank water to rehydrate. They were then divided in two equivalent groups of eight subjects (Table 1). Subjects of the studied group were previously trained to relax by performing DB and concentrating on their breath. These athletes spent 1 h (6:30–7:30 pm) relaxing performing DB in a quiet place. The other eight subjects, representing the control group, spent the same time sitting in an equivalent quite place. The only activity allowed was reading magazines. Lighting levels were monitored throughout the experiment and did not exceed 15 lux, a level well below that known to influence melatonin secretion [73, 74].

After the resting and DB periods, athletes consumed the same food and retired for sleeping at 10:00 pm. At 11:00 pm, all of them were sleeping.

We referred to the DB applied here as a relaxation technique. Instead of training the athletes with some form of meditation, we preferred DB because it is easy to learn and to perform and because it does not require any moral conviction that could generate psychologically adverse reactions. However, DB associated with a focused mind (in this case, awareness on the breath as specified in the methods section) can be considered a form of meditation such as focused meditation or others [75].

### 2.3. Oxidative Stress Determination

Oxidative stress was measured by performing the d reactive oxygen metabolites (d-ROMs) test [76, 77], which determines the plasma ROMs produced by ROS. The d-ROMs test is based on the concept that plasmahydroperoxides react with the transition metal ions liberated from the proteins in the acidic medium and are converted to alkoxy and peroxy radicals. These newly formed radicals are able to oxidize *N*,*N*-diethyl-para-phenylendiamine to the corresponding radical cation, and its concentration can be determined through spectrophotometric procedures (absorption at 505 nm). The d-ROMs test is expressed in U CARR (Carratelli units), where 1 U CARR = 0.08 mg H_2_O_2_ dl^−1^. Values higher than 300 U CARR indicate oxidative stress. ROMs were determined before starting the exercise (9:30 am), at the end of the exercise (6:00 pm), immediately after the DB periods (7:30 pm), at 2:00 am, and 24 h after the exercise (10:00 am of the following day).

### 2.4. Biological Antioxidant Potential Determination

The antioxidant defense status was assessed by determining the biological antioxidant potential (BAP test), which depends on the plasma levels of antioxidants. The BAP test is based on the ability of a coloured solution, containing a source of ferric (Fe^3+^) ions adequately bound to a special chromogenic substrate, to lose colour when Fe^3+^ ions are reduced to ferrous ions (Fe^2+^), which occurs when a reducing/antioxidant system is added. The ferric chloride reagent (50 *μ*L) is transferred into a cuvette containing the thiocyanate derivative reagent. The resulting colored solution is gently mixed by inversion and its absorbance is measured at 550 nm. Then, 10 *μ*L of plasma is added to the same cuvette, the solution is gently mixed, incubated in a thermostatic block for 5 min at 37°C, and its absorbance at 550 nm is remeasured [78, 79]. The BAP test results are expressed in *μ*moL Fe^2+^/liter of sample. Values higher than 2200 *μ*molLFe^2+^/liter are considered a normal BAP. d-ROMs and BAP tests were performed using apposite kits and dedicated instrumentation Free Radical Analytical System 4 (FRAS4, Health & Diagnostics Limited Co., Parma, Italy). Since the BAP test must be performed at least 3 h after food was last consumed, the BAP was determined before breakfast at 8:00 am, during the night at 2:00 am, and 24 h post-exercise (8:00 am).

### 2.5. Saliva Collection

The subjects abstained from alcoholic and caffeinated beverages from the beginning of the training session and were only allowed to drink water. Subjects washed their mouths with distilled water before salivary samples were obtained using the Bühlmann saliva collection device (Bühlmann Laboratories AG, Switzerland). Immediately after collection, the saliva samples were frozen and stored at –80°C until they were assayed for cortisol and melatonin concentrations.

### 2.6. Cortisol Assay

Salivary cortisol was determined before the exercise began (10:00 am), at the end of the exercise (6:00 pm), immediately after the DB period (7:30 pm), at 2:00 am, and 24 h after the exercise (10:00 am of the next day) using a commercially available EIA kit (Cortisol Express, Cayman Chemical Ann Arbor, MI, USA). Absorbance values were determined at 415 nm using a plate reader. Samples were assayed in triplicate.

### 2.7. Melatonin Assay

Salivary nocturnal melatonin was determined at 2:00 am using the Bühlmann Direct Saliva Melatonin Elisa (Bühlmann Laboratories AG, Switzerland). This assay is based on a melatonin biotin conjugate antibody, streptavidin conjugated to horseradish peroxidase and a tetramethyl benzidine (TMB) substrate. The product of the substrate was measured spectrophotometrically at 450 nm. The assay sensitivity range was 1–60.6 pg ml^−1^.

### 2.8. Statistical Analysis

The characteristics of the studied sample and the effects of DB were analyzed by two-way ANOVA with repeated measurements. A two-sided *t*-test (*post-hoc* comparisons) and the non parametric Wilcoxon-Mann-Whitney test were used for the comparison of numerical data across groups for each time point. A *P*-value < .05 was considered statistically significant. Statistics were compiled using Microsoft Excel and Winstat software. Changes in melatonin levels were analyzed by the two-sided *t*-test and the non-parametric Wilcoxon-Mann-Whitney test.

## 3. Results

### 3.1. Characteristics of the Studied Sample

Subjects were divided into two similar groups, as shown in Table 1. There were no statistical differences for age, height, weight, or km covered between the groups [*F*(1,62) = 0.023; *P* > .5].

### 3.2. Oxidative Stress Changes

**Figure 1:**
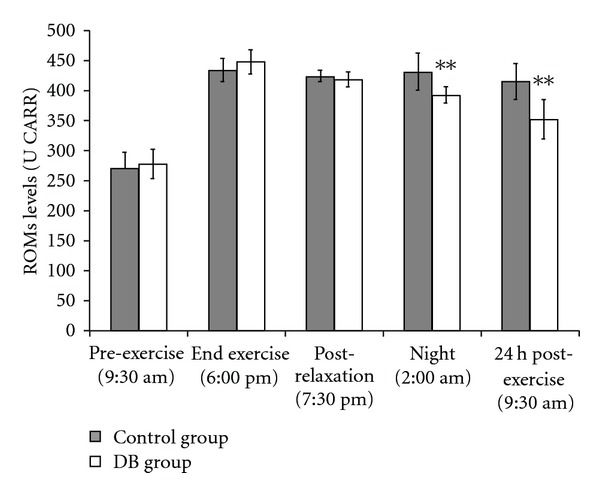
ROMs levels were determined at different times, before and after exercise. Athletes were divided in two equivalent groups of eight subjects. Subjects of the studied group spent 1 h (6:30–7:30 pm) relaxing performing DB and concentrating on their breath in a quiet place. The other eight subjects, representing the control group, spent the same time sitting in an equivalent quite place. Values shown are mean ± SD. **P < .01 DB versus control group.

As expected, the exercise induced a strong oxidative stress in athletes (Figure 1).

The ROMs levels were significantly increased after exercise compared to pre-exercise levels. All athletes had an elevation in ROMs in response to the training exercise, reaching particularly high levels of oxidative stress. The overall ANOVA revealed a significant DB effect [*F*(1,78) = 11.184; *P* < .01] and time effect [*F*(4,75) = 130.481; *P* < .01]. After completing the training exercise, there was a significant amount of variability between the ROMs levels of individual athletes, suggesting that each athlete has an individual response to oxidative stress. However, *post-hoc* comparisons confirmed that the mean level of ROMs in athletes of the DB group was significantly lower than the control-group athletes both at 2:00 am (*P* < .01 DB versus control group) and 24 h post-exercise (*P* < .01 DB versus control group). For the DB group, the increase in ROMs levels post-exercise compared to pre-exercise levels was 161.7% at 6:00 pm, 150.9% at 7:30 pm, 141.6% at 2:00 am and 126.8% 24 h post-exercise. For the control group, the increase in ROMs levels post-exercise compared to pre-exercise levels was 160.9% at 6:00 pm, 157.1% at 7:30 pm, 159.9% at 2:00 am and 154% 24 h post-exercise.

### 3.3. Biological Antioxidant Potential Changes

**Figure 2:**
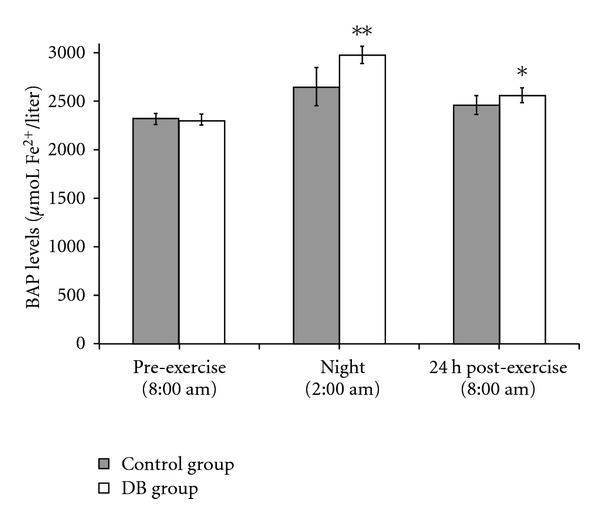
BAP levels were determined at different times, before and after exercise. Athletes were divided in two equivalent groups of eight subjects. Subjects of the studied group spent 1 h relaxing performing DB and concentrating on their breath in a quiet place. The other eight subjects, representing the control group, spent the same time sitting in an equivalent quite place. Since this test must be performed several hours after food ingestion, BAP levels were determined pre-exercise at 8:00 am before breakfast, at 2:00 am, and at 8:00 am 24 h post-exercise. Values shown are mean ± SD. *P < .05 DB versus control group. **P < .01 DB versus control group.

Figure 2 shows the BAP which significantly increased in both groups.

Again, a significant variation among the subjects was observed, but athletes of the DB group presented BAP levels significantly higher than the control group [*F*(1,46) = 21.001; *P* < .01]. This difference was more evident at 2:00 am (*P* < .01 DB versus control group, *post-hoc* comparisons) than 24 h post-exercise (*P* < .05 DB versus control group, *post-hoc* comparisons), where BAP began to return to basal levels. With respect to the pre-exercise values, for the DB group, the increase in BAP levels was 129.1% at 2:00 am and 111.1% at 24 h post-exercise.

For the control group, the increase was 114.2% at 2:00 am and 106.2% at 24 h post-exercise with respect to the pre-exercise values. ANOVA also revealed a significant time effect [*F*(2,45) = 91.587; *P* < .01].

### 3.4. Changes in Cortisol Levels

**Figure 3:**
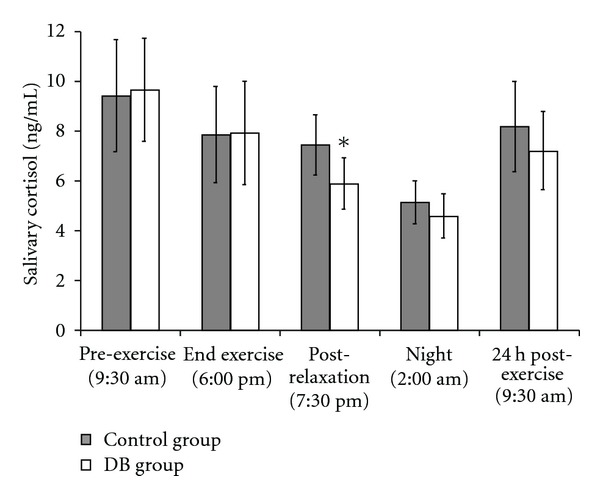
Salivary cortisol levels were determined at different times, before and after exercise. Athletes were divided in two equivalent groups of eight subjects. Subjects of the studied group spent 1 h (6:30–7:30 pm) relaxing performing DB and concentrating on their breath in a quiet place. The other eight subjects, representing the control group, spent the same time sitting in an equivalent quite place. Values shown are mean ± SD. *P < .05 DB versus control group.

ANOVA revealed a significant DB effect [*F*(1,78) = 4.028; *P* < .05]. As shown in Figure 3, significant differences between the groups were observed only at 7:30 pm, after the DB (*P* < .05 DB versus control group, *post-hoc* comparisons). At 2:00 am and 24 h post-exercise, cortisol levels were lower in athletes of the DB group, but differences were not statistically significant. In athletes of the DB group, the decrease in cortisol levels (07:30 p.m.) temporarily precedes the decrease in ROMs levels (2:00 am). It was not possible to determine the effects of exercise on cortisol levels, as hormone concentrations were determined at different times during its circadian rhythm. With respect to the pre-exercise values, for the DB group, cortisol values were 82.2% by 06:00 pm, 61.1% by 7:30 pm, 47.7% by 02:00 am and 74.7% 24 h post-exercise. For the control group, with respect to the pre-exercise values, values were 83.4% by 06:00 pm, 79.1% by 7:30 pm, 54.6% by 02:00 am and 86.9% 24 h post-exercise respect to the pre-exercise values. ANOVA also revealed a significant time effect [*F*(4,75) = 17.459; *P* < .01].

### 3.5. Changes in Melatonin Levels

**Figure 4:**
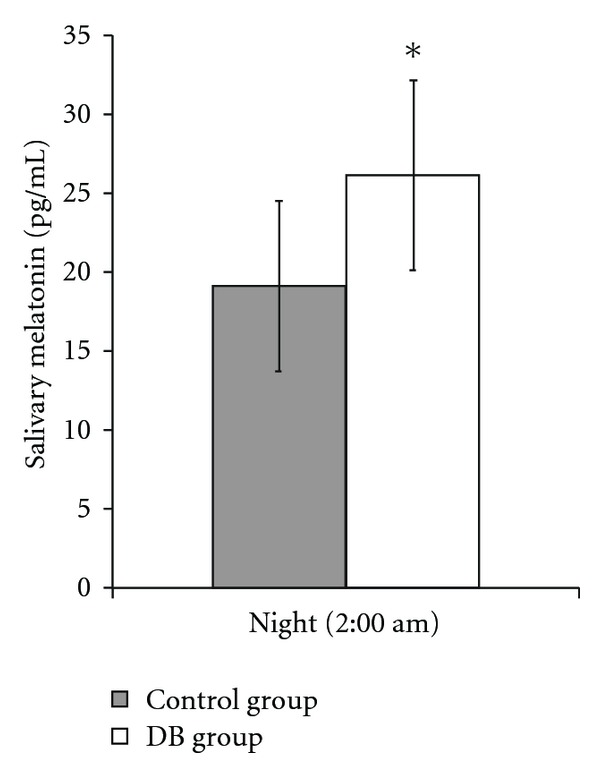
Salivary nocturnal melatonin levels variation after exercise. Values shown are mean ± SD. *P < .05 DB versus control group.

Figure 4 shows the differences in nocturnal melatonin levels between the two groups of athletes. Melatonin levels were significantly higher in athletes of the DB group (*P* < .05 DB versus control group). These data are congruent with the lower ROMs levels, with the higher BAP levels and with the lower cortisol levels at 7:30 pm.

## 4. Discussion

This study demonstrates that DB reduces the oxidative stress induced by exhaustive exercise. To our knowledge, this is the first study which explores the effect of DB on the stress caused by exhaustive physical activity.

It is known that cortisol inhibits enzymes responsible for the antioxidant activity of cells and that melatonin is a strong antioxidant. After the training exercise, athletes who underwent DB presented higher levels of BAP, which are congruous with the reduced levels of cortisol and ROMs and with the increased levels of nocturnal melatonin. As in our previous study [72], after exercise, we found an increase in BAP levels in both of the groups analyzed. However, the elevated levels of plasma antioxidant markers after exercise can be explained considering three processes: (i) the suspension of exercise decreases oxidant production, so antioxidant defense can return to normal levels; (ii) up-regulation of antioxidants and (iii) the mobilization of antioxidants from tissues to blood [80]. Beyond these mechanisms, these results also suggest that cortisol and melatonin levels could affect the modulation of antioxidant defenses and are relevant in determining the final level of oxidative stress. The decrease of ROS concentrations in subjects performing DB could be attributed to the reduced neuroendocrine response induced by relaxation.

**Figure 5:**
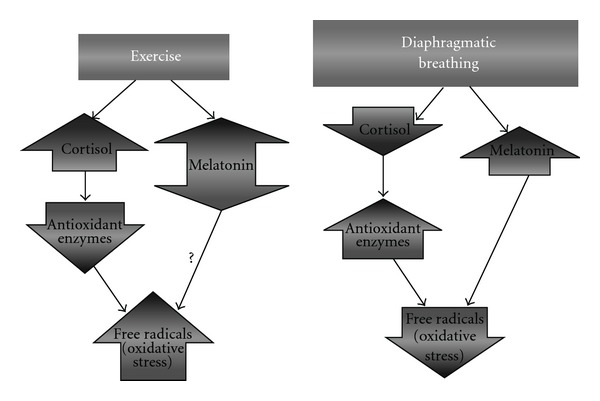
Modulation of oxidative stress by exercise and DB.

The rationale is as follows (Figure 5):

- intense exercise increases cortisol production;
- a high plasmatic level of cortisol decreases body antioxidant defenses;
- a high plasmatic level of cortisol correlates with a high level of oxidative stress;
- DB reduces the production of cortisol;
- DB increases melatonin levels;
- melatonin is a strong antioxidant;
- DB increases the BAP and
- DB reduces oxidative stress.

If these results are confirmed in other intense physical activity programs, relaxation could be considered an effective practice to significantly contrast the free radical-mediated oxidative damage induced by intense exercise. Therefore, similar to the way that antioxidant supplementation has been integrated into athletic training programs, DB or other meditation techniques should be integrated into many sports as a method to improve performance and to accelerate recovery. However, wider health implications can be accounted for the use of DB, as it can find applications in several pathologies. For example, the oxidative stress that occurs in the hyperventilation syndrome can be cured by learning DB. Hyperventilation, in fact, induces hyperoxia which is known to be related with oxidative stress [81, 82]. The hyperventilation syndrome affects 15% of the population and occurs when breathing rates elevate to 21–23 bpm as a result of constricted non-DB. DB can treat hyperoxia and its consequences acting by two synergic ways: restoring the normal breath rhythm and reducing oxidative stress mainly through the increase in melatonin production which is known for its ability to reduce oxidative stress induced by exposure to hyperbaric hyperoxia [83]. Moreover, Orme-Johnson observed greatly reduced pathology levels in regular meditation practitioners [84, 85]. A 5 years statistic of approximately 2000 regular participants demonstrated that Transcendental Meditation reduced benign and malignant tumors, heart disease, infectious diseases, mental disorders and diseases of the nervous system. Mourya et al. evidenced that slow-breathing exercises may influence autonomic functions reducing blood pressure in patients with essential hypertension [86]. Finally, there are also evidences that procedures which involve the control of the breathing can positively affect type 2 Diabetes [87], depression, pain [88], high glucose level and high cholesterol [89].

Our results contribute to explain these effects as oxidative stress may also play a role in the development of these pathologies [2–15]. The role of melatonin must also be emphasized. Beyond its antioxidant properties, melatonin is involved in the regulation of the circadian sleep-wake rhythm and in the modulation of hormones and the immune system. Due to its wide medical implications, the increase in melatonin levels induced by DB suggests that this breath procedure deserves to be included in public health improvement programs.

In this work, we explored the acute effects of DB, but these outcomes should also be investigated for longer periods, for which we would expect a more intense and beneficial response. For example, it is likely that expert practitioners who frequently utilize of DB could obtain a more significant reduction in oxidative stress and, perhaps, an improvement in exercise performance. Moreover, relaxation could also be improved by adding another relaxation method to the formula, for example music. In fact, Khalfa et al. [90] demonstrated that relaxing music facilitates recovery from a psychologically stressful task, decreasing the salivary cortisol.

Our results must also be discussed in light of the fact that cortisol has an ACTH-dependent circadian rhythm with peak levels in the early morning and a nadir at night. Athletes start to ride at 10:00 am and stop at 6:00 pm. The DB session started at 6:30 pm and stopped at 7:30 pm. It is probable that these results would be different if the time of physical activity and the DB session were changed. The same is true for melatonin. In fact, significant differences have been reported in melatonin secretion when exercises were performed at different times and under different light conditions [64–70]. We collected the saliva at 2:00 am, when a peak in melatonin must be expected. DB increased the levels of melatonin in athletes, and this correlates with lower oxidative stress (ROMs), with lower cortisol levels and with the higher antioxidant status (BAP) in these athletes.

The mechanism by which relaxation might induce an increase in melatonin levels is uncertain, and whether the melatonin increase is simply due to the cortisol decrease remains to be elucidated. Different mechanisms could be involved. Tooley et al. [46] speculated that meditation-reduced hepatic blood flow [91] could raise the plasma levels of melatonin. Alternatively, since meditation increases plasma levels of noradrenaline [92] and urine levels of the metabolite 5HIAA [93], a possible direct action on the pineal gland could be hypothesized, as melatonin is synthesized in the pineal by serotonin under a noradrenaline stimulus [94]. More likely, we suspect that the increase in melatonin levels determined in our experiment can be mainly attributed to the reduced cortisol levels. Actually, a relationship between cortisol and melatonin rhythms has been observed [95], indicating that melatonin onset typically occurs during low cortisol secretion. In addition, Monteleone et al. [96] found that exercise-induced increases in plasma cortisol preceded the lower night-time melatonin secretion, thus suggesting a connection between the metabolisms of these two hormones.

More studies are needed to clarify the link between cortisol and melatonin; however, due to the complexity of the pathways involved in maintaining homeostasis and in initiating the stress response, it is plausible that the relationship between the two hormones could be mediated by several mechanisms.

Overall, these data demonstrate that relaxation induced by DB increases the antioxidant defense status in athletes after exhaustive exercise. These effects correlate with the concomitant decrease in cortisol, which is known to negatively affect antioxidant defenses, and the increase in melatonin, a strong antioxidant. The consequence is a lower level of oxidative stress, which suggests that an appropriate recovery could protect athletes from long-term adverse effects of free radicals.

## Figures and Tables

**Table 1:** Characteristics of the sample studied.

Athletes	DB group				Control group				All athletes			
	Age (years)	Height (cm)	Weight (kg)	Kilometers covered	Age (years)	Height (cm)	Weight (kg)	Kilometers covered	Age (years)	Height (cm)	Weight (kg)	Kilometers covered
Mean	44.50	175.25	68.63	130.13	44.38	175.50	69.13	128.63	44.44	175.38	68.88	129.38
SD	2.32	6.94	4.52	5.01	2.12	8.34	6.31	5.60	2.16	7.52	5.66	5.19
